# Hematologic changes and splenic index on malaria mice models given *Syzygium cumini* extract as an adjuvant therapy

**DOI:** 10.14202/vetworld.2019.106-111

**Published:** 2019-01-21

**Authors:** Lilik Maslachah, Rahmi Sugihartuti, Retno Sri Wahyuni

**Affiliations:** 1 Department of Basic Veterinary Medicine, Veterinary Pharmacy Laboratory, Universitas Airlangga, Surabaya, Indonesia; 2 Department of Basic Veterinary Medicine, Clinical Pathology Laboratory, Faculty of Veterinary Medicine, Universitas Airlangga, Surabaya, Indonesia

**Keywords:** hematology, splenic index, *Syzygium cumini*, *Plasmodium berghei*

## Abstract

**Aims:**

This research aimed to determine the efficacy of *Syzygium cumini* L. as an adjuvant therapy on blood changes and splenic index of mice model malaria.

**Materials and Methods:**

Mice were infected intraperitoneally with 0.2 ml red blood cell (RBC) that contains 1×10^6^ *Plasmodium berghei*. 35 mice were divided into seven treatment groups: Group K0: Mice were not infected; K1: Mice were infected; K2: Mice were infected and given chloroquine; P1: Mice were infected and given *S. cumini* leaf extract; P2: Mice were infected and given chloroquine and also *S. cumini* leaf extract; P3: Mice was infected and given *S. cumini* stem bark extract; and P4: Mice were infected and given chloroquine and *S. cumini* stem bark extract. Treatment was given for 4 days 24 h post *-P. berghei* infection. 21^st^ day post-*P. berghei* infection, blood was taken from the heart for hematological examination, and the spleen was taken to examine the splenic index and also to measure the weight and length of the spleen. Hematological data and splenic index were analyzed by analysis of variance test, and if there is a difference, the test is continued by Duncan’s multiple range test with 5% level.

**Results:**

The K0 group has normal hemoglobin (HGB), RBC, and hematocrit (HCT) and significantly different (p<0.05) than other groups. HGB, RBC, and HCT of K1 group were under normal range, lowest, and significantly different (p<0.05) than other groups. Mean corpuscular volume and mean corpuscular HGB values of K2 groups showed a decrease. The number of leukocytes, lymphocytes, and monocytes of K1 groups was increasing and significantly different (p<0.05) with K2 and treatment group. The length, width, weight, and splenic index of K1 group were significantly different (p<0.05) with K0 group. K2 and treatment groups showed that the length and width of spleens were significantly different (p<0.05) with K1.

**Conclusion:**

The combination of chloroquine with leaf and chloroquine with stem bark extract of *S. cumini* as adjuvant therapy may increase the amount of erythrocyte; decrease the number of leukocytes, lymphocytes, and monocytes; and decrease the length, width, and splenic index on malaria mice models.

## Introduction

Malaria is still a public health problem in 107 countries until right now because malaria is still the fifth rank of infectious diseases in the world [1]. The control and treatment of malaria are more difficult recently because malaria parasites have been resistant to drugs and also the mosquitoes are resistant to insecticides. The development of treatment, prevention, and control of malaria is one of the substantial problems in the world. In 2012, approximately 600,000 children died, mostly from Sub-Saharan Africa [2].

The death of malaria infection is caused by very serious systemic complications such as hematological abnormalities, splenomegaly, and liver dysfunction. Hematological abnormalities during malarial infection are caused by high parasitemia in the patient; hematological abnormalities are also associated with endemicity, hemoglobinopathy, nutritional status, demographic factors, and immunity [3]. Malaria parasite infections may also induce splenic responses characterized by splenomegaly. The size of spleen is used as a tool for determining the intensity of malaria transmission in endemic areas [4]. During the erythrocytic stages of malaria infection, spleen is an important organ in the immune response. Elimination of infected erythrocytes through modulating the immune response and spleen remodeling, resulting in stringent splenic retention of rings and uninfected erythrocytes reduce the risk of cerebral malaria so that severe malaria does not occur [5].

The World Health Organization recommends the use of artemisinin in combination with other antimalarial drugs, but it has been reported that there was resistance to artemisinin monotherapy and artemisinin-based combination therapies combination on clinical cases in Cambodia [6]. In addition, there is a decrease in efficacy of antimalarial drugs that currently used, so it is important to develop an adjuvant therapy that can work on specific biologic pathways in the pathophysiology of malaria. Adjuvant therapy that can be used in severe malaria is immune system modulator preparations, antioxidants, anticoagulants, and agents that have anti-seizure activity [7]. The results showed that the use of matrix metalloproteinase (MMP) inhibitor was able to increase the survival of mice in cerebral malaria, and dexamethasone was able to decrease inflammation in murine malaria model with lung pathology. The use of quercetin flavonoids was able to block the induction of hemozoin for upregulation of MMP9, tumor necrosis factor-alpha (TNF-α), and interleukin-1beta [8-10]. *Syzygium cumini* contains anthocyanin, ellagitannin, ellagic acid, phenolic, flavonoids, and vitamins so that it has a high antioxidant activity. This plant is one of the medicinal plants which is easy to be found in Indonesia [11,12]. Results of research conducted by Zhang *et al.*, [13] showed that *S. cumini* has radical scavenging activity and strong antioxidant.

The research was aimed to determine the efficacy of *S. cumini* L. as an adjuvant therapy on hematological changes (red blood cells [RBC] and white blood cells [WBC]) and splenic index in mice model malaria.

## Materials and Methods

### Ethical approval

This study has obtained approval by certificate no 722-KE from Animal Care and Use Committee on Veterinary Medicine, Universitas Airlangga, Surabaya, Indonesia.

### Parasite, host, and drugs that used in this research

The parasite that is used to infect the mice is *Plasmodium berghei* ANKA strain. The mice used were male Swiss albino mice with 20-30 g weight and 2.5 months old, and obtained from Veterinary Farma Surabaya (Pusvetma) Center. Antimalarial drug used chloroquine pro analysis (PA) from Sigma Chemical Co. The chloroquine dose used 25 mg/kg body weight (BW) mice as a therapeutic dose. This drug was administered daily for 4 days [14]. The leaves and stem bark of *S. cumini* are obtained from Kediri city of East Java, Indonesia, and identified in the laboratory of Purwodadi Botanical Garden, Pasuruan. *S. cumini* dose was 600 mg/kg BW [15].

### Infection dose of *P. berghei* in mice

Mice were infected with 0.2 ml RBC containing 1×10^6^ *P. berghei* parasites intraperitoneally. To find out the infection which has occurred in mice, daily microscopic examination of erythrocyte was performed with a thin blood smear taken from the vein of the tail and stained with Giemsa 20% [16]. Calculation of the dose of parasitic infection was determined by counting the number of parasites from the thin blood smear that stained with Giemsa and then calculated the number of parasites per number of erythrocytes. The next step is calculating the number of erythrocytes by diluting the blood using PBS solution in Eppendorf 0.5 ml. Then, these diluted blood erythrocytes were calculated using the improved Neubauer counting chamber. The number of parasitic doses is obtained from multiplying by the number of parasites with the number of erythrocytes that have been calculated and converted to per ml.

### Preparation of leaf and stem bark of *S. cumini*

The leaves and stem bark of *S. cumini* are dried, after that, it was crushed into small pieces (simplicia). Simplicia was extracted with PA methanol and maceration for 3×24 h. The filtrate was evaporated using a Rotary Evaporator at 40-50°C with low pressure. The extraction results are stored on the desiccator until ready for use [17].

### Treatment of the experimental animals

A total of 35 mice were randomly divided into seven treatment groups, and each group consists of 5 mice. Details of each group are as follows:

Group K0: Mice were only given drug solvent and not infected; K1: Mice were infected and given drug solvent; K2: Mice were infected and given chloroquine 25 mg/kg BW; P1: Mice were infected and given *S. cumini* leaf extract 600 mg/kg BW; (P2): Mice were infected and given chloroquine 25 mg/kg BW and also given *S. cumini* leaf extract 600 mg/kg BW; (P3): Mice were infected and given *S. cumini* stem bark extract 600 mg/kg BW; and (P4): Mice were infected and given chloroquine 25 mg/kg BW and also given *S. cumini* stem bark extract 600 mg/kg BW.

Treatment was given for 4 days since 24 h after per oral infection. After 21 days post-infection, mice were anesthetized with ketamine (Sigma) and then thoracotomy, blood samples were taken from the heart (1 ml) using a Tuberculin Syringe and collected in a vial that has been given anticoagulation for hematologic examination using automated blood analyzer SYSMEX XT 4000i, and spleen was taken to examine the splenic index and also measure the weight and length of spleen.

### Examination of weight, length, and splenic index

Previously, the weight of the mice was measured, after that the mice were injected with ketamine IM. The abdominal cavity was opened, and the spleen was taken and weighed using an analytical scale and then measured the length and width of the spleen using a ruler on a millimeter scale. According to Gluhcheva *et al*. [18], the splenic index was calculated using the lymph index equation = (weight of spleen organ of mice)/bw of mice.

### Statistical analysis

The data of hematology observation of blood change and lymph index were processed using analysis of variance using SPSS System 17.0 then followed by Duncan’s multiple range test with 5% level.

## Results

### Hematological results of RBC

The results of statistical tests of mean hemoglobin (HGB), RBC, hematocrit (HCT), mean corpuscular volume (MCV), mean corpuscular HGB (MCH), and MCHGB concentration is presented in Table-1. The mean value of HGB in the non-infected control group (K0) was normal (15.45 g/dl; the normal range is 13.4-15.8 g/dl) HGB levels in the K0 group were significantly different from the other treatment groups, namely K1, K2, P1, P2, P3 and P4 (p<0.05). Among these groups, there was no significant difference between the K2 group and P2 and P3, and between P1 and P4 (p>0.05).

**Table-1:** Mean and standard deviation of RBC hematology in control and treatment groups.

Mice groups	Parameter of RBC hematology						

	HGB (g/dL)	RBC (10^6^/µL)	HCT (%)	MCV (fL)	MCH (pg)	MCHC (g/dL)	RDW-CV (%)
K0	15.45±0.50^d^	10.56±0.27^d^	51.40±2.55^c^	48.10±3.39^a^	14.60±0.53^b^	30.30±0.49^a^	23.42±0.70^a^
K1	11.12±0.64^a^	8.09±0.33^a^	36.80±3.88^a^	45.52±4.95^a^	13.75±0.71^ab^	30.37±1.80^a^	25.92±3.51^b^
K2	12.25±0.12^b^	8.90±0.89^b^	39.70±0.98^ab^	45.37±4.91^a^	13.87±1.07^ab^	30.97±1.06^a^	25.85±0.23^b^
P1	13.12±0.41^c^	9.14±0.36^bc^	42.57±2.13^b^	46.97±2.57^a^	14.40±0.57^ab^	30.67±0.45^a^	21.80±0.66^a^
P2	12.80±0.29^bc^	9.75±0.36^c^	42.55±2.13^b^	43.72±1.31^a^	13.25±0.36^a^	30.20±0.87^a^	23.97±0.59^a^
P3	12.32±0.45^b^	9.15±0.53^bc^	40.15±3.70^ab^	43.82±2.47^a^	13.52±0.67^ab^	30.85±1.88^a^	24.67±3.51^ab^
P4	13.07±0.36^c^	9.14±0.62^bc^	41.26±1.03^b^	45.42±3.39^a^	14.37±0.77^ab^	31.67±0.65^a^	23.30±126^ab^

Different superscripts on the same column show a significant difference at significant level of 0.05%, RBC=Red blood cell, HGB=Hemoglobin, HCT=Hematocrit, MCH=Mean corpuscular hemoglobin

The amount of RBC in the K0 group was significantly different from the number of RBCs in the entire treatment group (p <0.05). However, there was no significant difference in the number of RBCs in the treatment groups K2, P1, P3, and P4 and between the treatment groups P1, P2, P3 and P4 (p> 0.05).

The average of HCT/PCV level in the K0 group was significantly different from all treatment group in this study (p<0.05). However, there was no significant difference between treatment groups K1, K2 and P3 and between treatment groups K2, P1, P2, P3 and P4 (p>0.05).

### Hematology result of WBCs

The mean number of leukocytes (WBC) increased in the K1 group infected with *P. berghei* and was significantly different (p<0.05) when compared with the infected group treated with chloroquine (K2 group) and the group treated with the combination of *S. cumini* leaf and stem bark extract with chloroquine (P2 and P4 groups). The average number of leukocytes in K2, P1, P2, P3, and P4 was still within a normal range between 8.00 and 11.8 (103/m^3^). The average platelet value was still within normal limits. The highest lymphocyte values are in the K1 group and were significantly different from K0, P2, and P4 and did not differ significantly with K2, P1, and P3 with a normal range limit of 6.03-8.90. The highest average monocyte count in the K1 group was significantly different (p<0.05) with K0, K2, and P4 and was not significantly different (p>0.05) with P1, P2, and P3. The average number of neutrophils showed varied results among the treatment groups. The treatment groups K0, K2, and P4 do not show significant differences. Similarly between treatment groups K2, P1, P2 and P3, and between treatment groups K1, P1, P2 and P3 (p>0.05). Significant differences were seen in the K0 group with K1, P1, P2 and P3 (p <0.05) Hematologic data of WBC are shown in Table-2.

**Table-2:** Mean and standard deviation of WBC hematology in control and treatment groups.

Mice group	Parameter of WBC hematology							

	WBC	PLT	Lymph	Lymph (%)	Mono	Mono (%)	Neut	Neut (%)
K0	4.33±0.17^a^	1322.25±35.34^ab^	3.60±0.11^a^	83.40±0.66^abc^	0.27±0.02^a^	6.20±0.29^a^	0.35±0.04^a^	8.07±0.17^b^
K1	13.22±6.35^c^	1551.50±451.38^b^	10.33±4.83^c^	79.87±6.95^ab^	2.24±2.02^c^	16.75±8.83^c^	0.46±0.44^a^	1.95±3.90^a^
K2	8.41±0.56^ab^	1432.75±21.09^ab^	7.08±0.20^bc^	88.37±2.00^c^	0.76±0.06^ab^	9.22±0.51^ab^	0.35±0.04^a^	2.07±2.39^a^
P1	10.59±1.14^bc^	1245.50±99.37^ab^	8.29±0.74^bc^	79.37±1.82^ab^	1.70±0.51^bc^	16.15±4.10^bc^	0.35±0.30^a^	3.22±3.80^a^
P2	8.17±0.30^ab^	1127.50±157.55^a^	6.17±0.33^ab^	76.07±2.36^a^	1.36±0.19^abc^	16.22±2.39^bc^	0.55±0.13^a^	6.90±0.90^ab^
P3	11.03±0.51^bc^	1097.25±295.11^a^	8.83±0.41^bc^	80.92±3.84^abc^	1.70±0.59^bc^	15.12±4.66^bc^	0.36±0.26^a^	2.52±3.17^a^
P4	7.87±2.48^ab^	1156.75±213.69^a^	6.62±2.28^b^	85.15±9.55^bc^	0.46±0.20^ab^	6.63±4.18^a^	0.66±0.36^a^	5.92±4.31^ab^

Different superscripts on the same column show a significant difference at significant level of 0.05%, WBC=White blood cell, PLT=Platelet

### Measurement results and splenic index

The result of length, width, weight, and splenic index measurement in treatment group infected with *P. berghei* showed an enlarged lymph organ and significantly different (p<0.05) with uninfected control group (K0). The length and width of the spleen in the K1 group which infected with *P. bergei* were significantly different from the K2 group that infected and treated with chloroquine, However, P1, P2, P3, and P4 showed no significant different (p>0.05) with K2. spleen data are shown in Table-3.

**Table-3:** Mean and standard deviations of length, width, weight, and splenic index of mice in the control and treatment groups.

Mice group	Length (cm)	Width (cm)	Weight (g)	Splenic index
K0	0.82±0.14^a^	0.22±0.12^a^	0.21±0.11^a^	0.008±0.002^a^
K1	3.07±0.22^c^	0.65±0.10^c^	0.44±0.14^c^	0.016±0.003^c^
K2	2.20±0.62^b^	0.45±0.19^b^	0.30±0.10^bc^	0.011±0.001^bc^
P1	2.65±0.26^bc^	0.57±0.09^bc^	0.37±0.08^bc^	0.013±0.001^c^
P2	2.42±0.38^bc^	0.50±0.11^bc^	0.34±0.10^bc^	0.013±0.003^bc^
P3	2.37±0.47^b^	0.60±0.08^bc^	0.31±0.04^bc^	0.013±0.002^bc^
P4	2.47±0.30^bc^	0.55±0.10^bc^	0.27±0.07^b^	0.009±0.002^b^

Different superscripts on the same column show a significant difference at significant level of 0.05%

## Discussion

### RBC hematology

The normal range of HBG, erythrocytes, and HCT in non-infected control group due to the mice under normal circumstances and the erythrocytes are not damaged. In the group that infected by *P. berghei* and was not treated and the infected group that treated with chloroquine, leaf, stem bark of *S. cumini*, and its combination, the HGB values, erythrocytic cell count, HCT mean, MCV, and MCH values showed a decrease below the normal range, and the lowest is in K1 which was different from the other treatment groups.

This suggests that Plasmodium infections cause erythrocyte hemolysis. The removal of infected and uninfected erythrocyte cause erythropoiesis in the body which becomes ineffective;, this is caused by the abnormally high levels of TNF that has effect on ineffective erythropoiesis [19].

*In vitro* and *in vivo* studies show pro-inflammatory cytokines including interferon y, TNF-α, and macrophage migration inhibitory factor, as well as Plasmodium products (hemozoin) which play a role in the pathogenesis of malarial anemia [20]. Hemozoin was produced from HGB digestion by Plasmodium and induces macrophage for the secretion of pro-inflammatory cytokines and other mediators that inhibit the effects of erythropoiesis [21,22]. Plasmodium hemozoin products play a role in erythropoiesis resistance, low reticulocytosis, and malarial anemia by inhibiting Epo-induced proliferation from erythroid precursors [23].

Malarial anemia due to Plasmodium infection may increase immunoglobulin G autoantibodies levels against non-infected RBC (nRBCs), and its deposition on the surface of nRBCs can decrease red cell deformability and improve erythrophagocytosis [24]. The results of this study in the treatment group, Hb levels, the number of erythrocytes, and levels of premature ventricular contractions are below normal, and it is due to hypochromic microcytic anemia because MCV and MCH values are below normal. The results of this experimental animal model were consistent with the research that occurred in humans infected with *Plasmodium falciparum* 71% of anemic patients having hypochromic microcytic anemia [19].

In the treatment groups (P1, P2, P3, and P4), the number of erythrocytes were within normal range. The antimalarial effects of chloroquine and antioxidants containing *S. cumini* leaf extracts cause proliferative resistance of *P. berghei*. It happened through the inhibition of hemozoin formation as well as through the parasite protease inhibition that involved in the degradation of HGB [25]. The decrease of proliferation in *P. berghei* infection in mice treated with chloroquine and combination with antioxidants can increase superoxide dismutase activity and decrease lipid peroxidation [26]. The combination of antimalarial therapy with antioxidants can counter the pathological damage due to oxidants and decrease the proliferation of parasites [27]. Previous research state that leaves, stems, and fruit of *S. cumini* have antioxidant and anti-inflammatory activity [28]. The value of IC50 leaves of *S. cumini* is 12.84 ppm, so it is potentially developed as an antioxidant [29]. Another research showed a positive association of antioxidant potential, the ability to reduce free radical and phenolic compound content on *S. cumini* leaf extract [30].

### WBC hematology

Increased WBC, the number of lymphocytes, and the number of monocytes in the K1 group infected with *P. berghei* were not treated. The number of WBC, lymphocytes, and monocyte were decreased in the K2 group as well as in P2 and P4 group. These results suggest that WBC elevation in infection by Plasmodium may stimulate the immune system as a physiological response of the body to malaria infection because the WBC has a role in against infection When an infection occurs, the body will respond by phagocytosis of infectious agent and stimulate increased production of immune cells to produce antibodies. The decrease in WBC in the infected and antimalarial-treated groups and the combination with the extract showed that the treatment was able to fight infection [31].

Lymphocytes as primary effector cells have a very important role in the immune system. Increased lymphocytes show the mechanism of body defense against *P. berghei* infection [31]. Monocytes are phagocytes. The increase in the number of monocytes suggests a body’s immune response to accelerating the activity of Plasmodium protozoa phagocytosis. Monocytes play an important role in the production, mobilization, and regulation of immune-effector cells, also contributing to infection elimination [32]. The reduction of lymphocytes and monocytes in the treatment of antimalarials and its combinations with the extracts due to phytochemicals glycoside, phenol, tannin, saponins, flavonoids, and ellagic acid in *S. cumini* stem bark extracts have antioxidant effects. The antioxidant effects of phytochemicals in mice infected with *P. berghei* were able to increase the antioxidant enzyme superoxide dismutase and catalase and decrease the concentration of malondialdehyde to improve hematologic parameters [33].

### Measurement and splenic index

The result of measurement of length, width, weight, and splenic index in treatment group infected with *P. berghei* shows the enlargement of lymph organs. Lymph works as an effector against malaria infection, especially in protective immunity against infections of the blood [34]. The total number of spleen cells increased during high parasitemia and then decreased at lower levels. Splenomegaly in malarial infection is associated with the expansion of the white pulp and the red pulp due to increased follicle size, that caused by hematopoietic reaction. Increased macrophage occurs due to the process of erythrophagocytosis. Macrophage in the red pulp lymph plays an important role in removing plasmodium that infects RBC from circulation [35].

Plasmodium infections in RBC may lead to complex pathophysiology, and rapid growth of Plasmodium will increase the production of reactive oxygen species causing an imbalance between plasma oxidants and host antioxidant systems leading to oxidative stress [36]. The administration of chloroquine antimalarial drug and leaf and stem bark extract of *S. cumini* and its combination can decrease the length, width, and splenic index. This shows the effects of antimalarials, and antioxidant content in leaf and stem bark extract of *S. cumini* as phenol compounds contained in the leaves include caffeine acids, chlorogenic acid, ellagic acid, ferulic acid, and gallic acid. The leaves are also contained tannins and essential oil terpenes [37]. *S. cumini* stem bark contains flavonoids, polyphenols, acetyl oleanolic acid, tannins, gallic acid, ellagic acid, quercetin, isoquercetin, kaempferol, myricetin, flavonol, glycosides, saponins, triterpenoids, and anthocyanins [38].

Research conducted by Jayachandra and colleague [39] showed that the total phenolic content was 580.23±3.03 mg/g, tannin content was 534±4.03 mg/g, while the flavonoid content was 315.42±4.52 mg/g. The methanolic and aqueous extracts of bark were screened for antioxidant activity using nitric oxide scavenging activity method. Antioxidant activity is caused by the flavonoid and polyphenol content [40]. The combination of deferoxamine administration as an iron chelator that has anti-plasmodial activity with ellagic acid as an antioxidant in *Plasmodium yoelii* infection produces an antimalarial additive effect [41]. Decreased lesions are reversible in the spleen following a decrease in parasitemia through apoptosis as a defense mechanism of spleen against malaria infection [34].

## Conclusion

The combination of chloroquine with leaf and stem bark extract of *S. cumini* as adjuvant therapy may increase the number of erythrocytes; decrease the number of leukocytes, lymphocytes, and monocytes; and decrease the length, width, and splenic index of malaria in mice models.

## Authors’ Contributions

LM designed the research, supervised the research, and compiled the manuscript; RS and RSW helped to analyze blood and spleen data. All authors have read and approved the final manuscript.
